# Septin and Ras regulate cytokinetic abscission in detached cells

**DOI:** 10.1186/s13008-019-0051-y

**Published:** 2019-08-21

**Authors:** Deepesh Kumar Gupta, Siamak A. Kamranvar, Jian Du, Liangwen Liu, Staffan Johansson

**Affiliations:** 1 Department of Medical Biochemistry and Microbiology, Biomedical Center, Uppsala University, Box 582, 751 23 Uppsala, Sweden; 2 First Hospital of Jilin University, Changchun, Jilin China

**Keywords:** Cytokinesis, Bi-nucleation, Regression, Septin, Ras, Adhesion

## Abstract

**Background:**

Integrin-mediated adhesion is normally required for cytokinetic abscission, and failure in the process can generate potentially oncogenic tetraploid cells. Here, detachment-induced formation of oncogenic tetraploid cells was analyzed in non-transformed human BJ fibroblasts and BJ expressing SV40LT (BJ-LT) ± overactive HRas.

**Results:**

In contrast to BJ and BJ-LT cells, non-adherent BJ-LT-Ras cells recruited ALIX and CHMP4B to the midbody and divided. In detached BJ and BJ-LT cells regression of the cytokinetic furrow was suppressed by intercellular bridge-associated septin; after re-adhesion these cells divided by cytofission, however, some cells became bi-nucleated because of septin reorganization and furrow regression. Adherent bi-nucleated BJ cells became senescent in G1 with p21 accumulation in the nucleus, apparently due to p53 activation since adherent bi-nucleated BJ-LT cells passed through next cell cycle and divided into mono-nucleated tetraploids; the two centrosomes present in bi-nucleated BJ cells fused after furrow regression, pointing to the PIDDosome pathway as a possible mechanism for the p53 activation.

**Conclusions:**

Several mechanisms prevent detached normal cells from generating tumor-causing tetraploid cells unless they have a suppressed p53 response by viruses, mutation or inflammation. Importantly, activating Ras mutations promote colony growth of detached transformed cells by inducing anchorage-independent cytokinetic abscission in single cells.

**Electronic supplementary material:**

The online version of this article (10.1186/s13008-019-0051-y) contains supplementary material, which is available to authorized users.

## Background

Normal adherent cells require signals generated from integrin contacts with the extracellular matrix for their survival and proliferation [1, 2], presumably as control mechanisms to prevent growth of detached or miss-located cells. This control is circumvented in malignant tumors and therefor “anchorage-independent growth” is commonly used as an in vitro marker for malignantly transformed cells. For proliferation, non-transformed cells need signals from integrins to pass the G1-S transition checkpoint [3] of the cell cycle and to execute abscission at the end of mitosis [4–6]. The adhesion-dependence for cytokinesis may help to protect against proliferation of detached cells that have a suppressed G1/S checkpoint, e.g. due to virus infection or mutation [7]. However, it may also have negative effects since blocked cytokinesis will result in bi-nucleated cells, which potentially can generate oncogenic aneuploid cells [8].

Cytokinesis in animal cells starts in anaphase by the formation of an actomyosin contractile ring which is connected to the plasma membrane via several proteins, including anillin and septin filaments [9, 10]. The contractile ring will cause plasma membrane ingression and compress the mitotic spindle into a dense bundle of antiparallel microtubules carrying associated kinases, motor and adaptor proteins which will form the structure called midbody (MB) [11]. The MB will mature by the stepwise recruitment of additional proteins, and eventually ESCRT III-dependent abscission of the intercellular bridge (ICB) will occur close to the MB at a site specified by septin rings [12, 13]. The actual membrane fusion mechanism is still not understood, but the polymerization of the ESCRT III subunit CHMP4B is known to bring the two opposing membranes close to each other. Failure in the cytokinesis process can cause regression of the cleavage furrow and the generation of bi-nucleated cells [14].

Our previous studies showed that the cytokinesis is halted in non-transformed detached fibroblasts and epithelial cells at a late stage of the process; specifically, the binding of ALIX to the major MB protein CEP55 is blocked and thereby also the subsequent ALIX-dependent recruitment of ESCRT III components to the MB is prevented [4]. In spite of the uncompleted cytokinesis the detached cells enter a new round of the cell cycle, in which the midbody is dissolved early in G1 [13]. However, the septin rings were found to remain longer at the ICB and thereby prevent the attached plasma membrane from regressing. If such cells are allowed to re-adhere to a fibronectin surface, most of them, but not all, will divide in the absence of MB by traction force (“cytofission”) [15]. Identification of the conditions and mechanisms that determine whether regression will happen or not is important to understand the fate of detached cells in the body and their possible contribution to tumor formation and/or progression.

Oncogenic Ras mutations are considered to provide cells with the ability to grow anchorage-independently, a view based on the promotion of colony growth in soft agar by Ras. However, this assay runs over several days and it does not provide information on whether a colony started from a single cell, from a group of cells, if the growth actually was independent of integrin signals, or if the cell(s) got integrin signals after formation of extracellular matrix (“pseudo-anchorage-dependent growth” [6]). Thus, it is unclear if Ras mutants induce passage of the G1-S checkpoint and cytokinesis by replacing integrin signals or if they promote pseudo-anchorage-independent growth. In order to directly study adhesion-independent cytokinesis it is necessary to analyze single cells. Interestingly, active Ras was in one study reported to induce cytokinesis in non-adherent human fibroblasts, as analyzed by FACS [16]. Because of the difficulty in analyzing bi-nucleated cells at the cytokinesis stage using FACS without breaking the narrow cytoplasmic bridge [17, 18] uncertainty still remains regarding the ability of oncogenic RAS to promote ESCRT III-mediated abscission in detached cells. In the present study we aimed to clarify this important issue, as well as to determine the fate of bi-nucleated cells in the following cell cycle and its dependence on p53.

## Results

We have previously shown that the cytokinesis process is blocked in detached non-transformed cells at the step of ALIX recruitment to CEP55 in the MB [4]. Here we analyzed the effects of the transforming viral protein LT and oncogenic HRas mutation on the cytokinesis of non-adherent cells using the human fibroblast cell lines BJ, BJ-LT, and BJ-LT-Ras. Furthermore, the fate of cytokinesis-blocked cells in the following cell cycle was investigated under adherent and non-adherent conditions. The procedures used for these studies are illustrated in Additional file 1: Figure S1.

### Active Ras, but not the LT protein, promotes abscission in detached cells

#### Analysis of MB markers

First the cytokinesis process was analyzed by immune-staining for ALIX and CHMP4B, markers for the adhesion-dependent step and the final abscission, respectively; Aurora B, CEP55, or α-tubulin were used as general MB markers since they are present in this structure from early stages to the abscission. Similar to our previously published results for BJ cells [4], a majority of BJ-LT cells (75%) isolated in M-phase by shake-off had reached cytokinesis and formed a MB after 1 h of adhesion to fibronectin (Additional file 2: Figure S2A, C), and abscission was completed in most cells within 2-3 h after the plating (Additional file 2: Figure S2D, Additional file 3: Movie S1). The isolated mitotic BJ-LT-Ras cells which were plated on fibronectin progressed to cytokinesis with similar rate as BJ-LT (Additional file 2: Figure S2B, C) and completed cytokines somewhat faster, 60% dividing within 2 h (Additional file 2: Figure S2D, Additional file 4: Movie S2). Also the mean overall cell cycle time was shorter for BJ-LT-Ras than for BJ-LT (Additional file 2: Figure S2E).

**Fig. 1:**
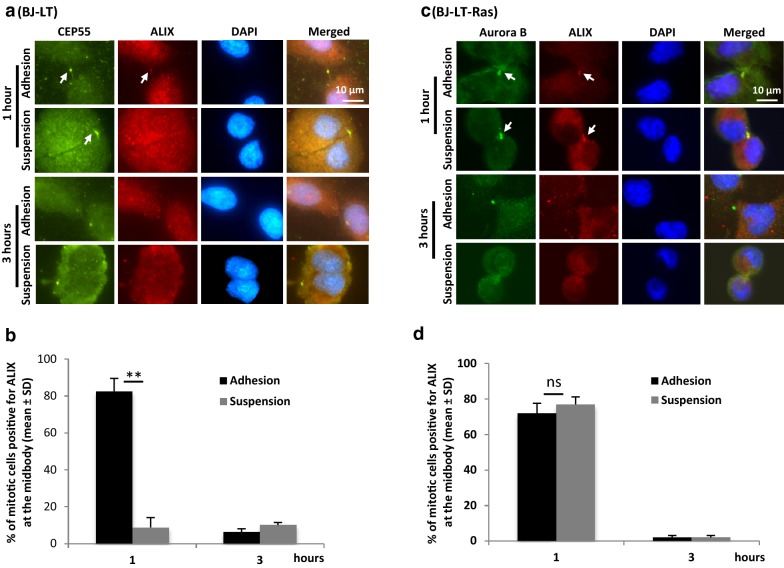
Active Ras, but not SV40 LT protein, promotes the recruitment of ALIX to the MB in detached cells. Mitotic BJ-LT (**a**, **b**) and BJ-LT-Ras (**c**, **d**) cells were isolated and cultured for the indicated time periods in suspension or on fibronectin. **a**, **c** Representative immunofluorescence micrographs illustrating the cells immunostained for Aurora B (green; MB marker), CEP55 (green; MB marker) and ALIX (red). Nuclei were stained with DAPI (blue). **b**, **d** Mean% ± SD of the number of cells having ALIX at the MB. After 3 h no midbody is detected because the cells have entered G1; in the adherent cells abscission has been completed at this time point

When the mitotic BJ-LT cells instead were kept in suspension, ALIX was not located at the MB at any time point and the cells did not divide; after 3–4 h in suspension the midbody was no longer detectable as previously reported for BJ cells [13] (Fig. 1a, b). In contrast to BJ [4] and BJ-LT cells, a similar number of the BJ-LT-Ras cells (77%) were positive for ALIX in the MB after incubation for 60 min under non-adherent conditions as on fibronectin (Fig. 1c, d). Furthermore, microtubules were densely packed in the ICB and CHMP4B became localized at the MB (Additional file 5: Figure S3C, D), features not found in non-adherent BJ [4] and BJ-LT cells (Additional file 5: Figure S3A, B).

**Fig. 2:**
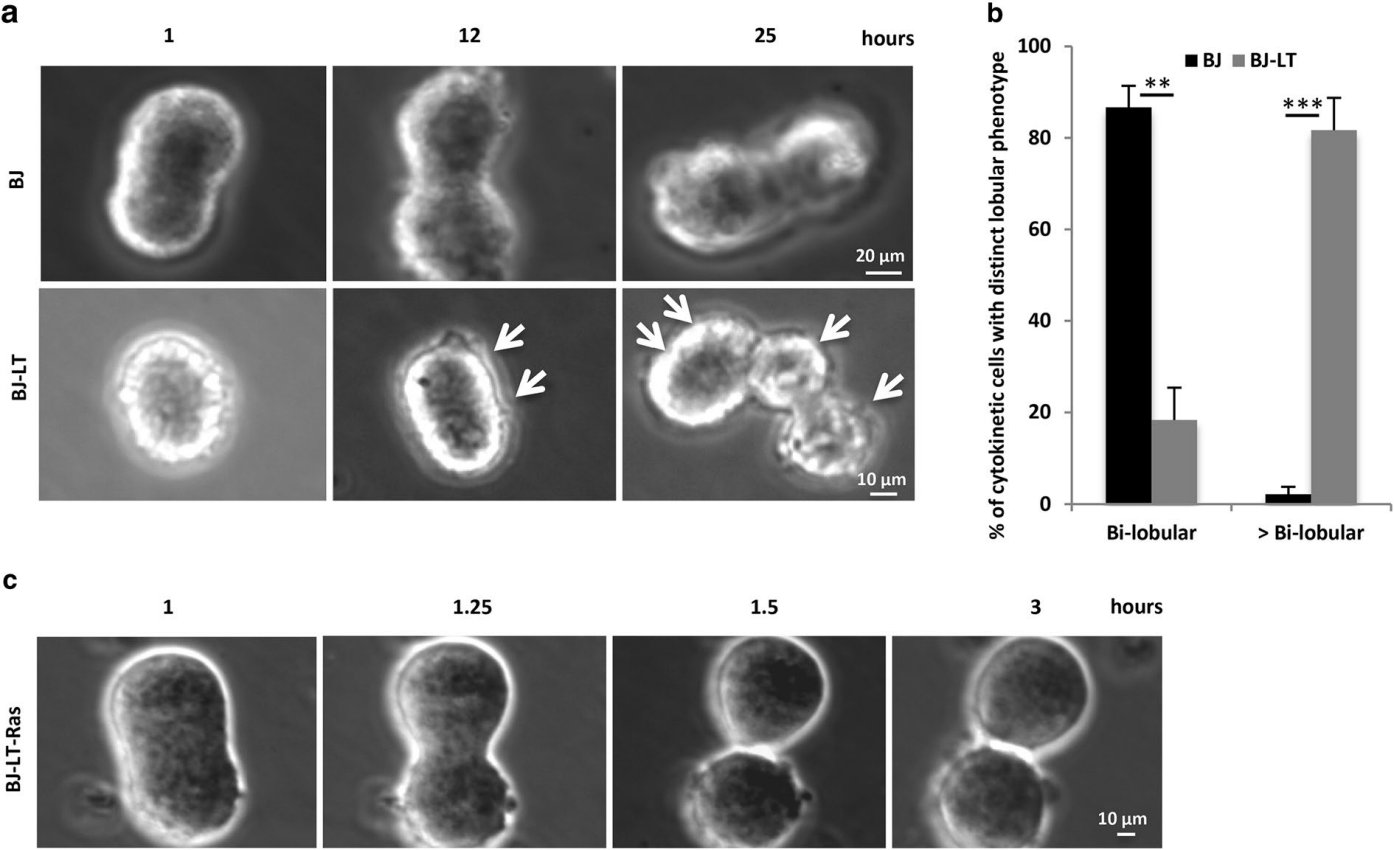
LT and active Ras induce different morphologies in the detached cells. M-cells were isolated and cultured in suspension for live-cell imaging. Bright field micrographs from representative time-lapse movies of single **a** BJ and BJ-LT cells, and **c** BJ-LT-Ras cell show the progression of cytokinesis at the indicated time points. **a** In the upper panel, the BJ cell shows a bi-lobular feature throughout the 25-h period. In the lower panel, the BJ-LT cell was initially bi-lobular and became tetra-lobular after 25 h as marked by the white arrows, due to progression of the cell cycle without completing cytokinesis. **b** Mean% ± SD of the number of cells having two or more nuclei after 25 h. **c** The BJ-LT-Ras fibroblast may have completed abscission after 1.5 h as indicated by the apparently continuous membrane between the two daughter cells, but they remain associated since they cannot migrate apart in suspension

To analyze if the passage of the critical CEP55-ALIX step led to completion of abscission in non-adherent BJ-LT-Ras cells, the cells were followed by time-lapse microscopy and compared with BJ and BJ-LT cells. In agreement with our previous studies [4, 6, 15], M-phase BJ cells in suspension underwent karyokinesis and cleavage furrow ingression but failed to divide and remained as bi-lobular cells even after 25 h. Similarly, BJ-LT cells did not complete cytokinesis, but after 24–25 h, each of the two nuclei divided and the bi-lobular cells became tetra-lobular [Fig. 2a, b, Additional file 6: Movies S3 (BJ) and Additional file 7: Movie S4, Additional file 8: Movie S5 (BJ-LT)]. This result is consistent with expectations for cells completing a new round of the cell cycle due to suppression of the G1/S checkpoint by the LT protein while maintaining the ICB. In contrast, the movies of BJ-LT-Ras cells indicated that the cells indeed did divide in suspension, and did so with approximately similar kinetics as BJ-LT-Ras cells adhering to fibronectin (Fig. 2c, Additional file 9: Movie S6, Additional file 10: Movie S7). However, since the two new cells stayed in close contact with each other, the possibility that the narrow ICB remained could not be excluded with certainty and therefore additional approaches to answer the question was tested.

#### Analysis of septin-7

**Fig. 3:**
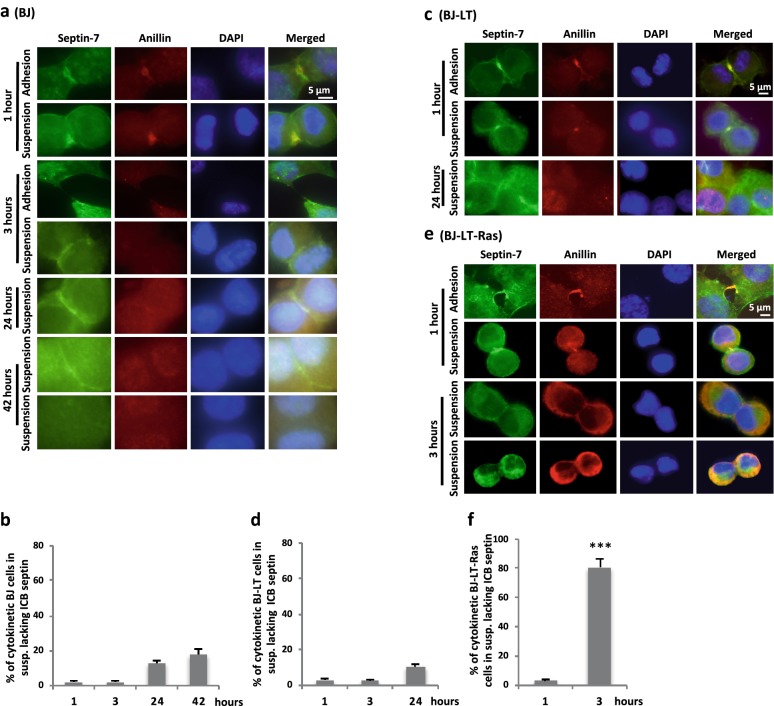
Septin marks the presence of an ICB. M-cells were isolated and either re-plated on fibronectin-coated dishes (adhesion) or non-adhesive dishes (suspension) for the indicated time points. Representative immunofluorescence images illustrating the localization of septin-7 (green) and anillin (red) in the adhesion and suspension conditions in **a** BJ cells, **c** BJ-LT cells, and **e** BJ-LT-Ras cells. Nuclei were stained with DAPI (blue). Note that for BJ and BJ-LT-Ras two examples are given at the 42- and 3-h time points, respectively, illustrating different septin-7 staining. Under adherent conditions, all three cell types had divided after 3 h (here shown only for BJ cells; see Additional file 5: Figure S3). **b**, **d**, **f** Mean% ± SD of the number of cells without septin-7 along ICB at the indicated time points for non-adherent BJ, BJ-LT, and BJ-LT-Ras cells. Difference significance: BJ-LT-Ras 3 h vs. BJ 3 h or BJ-LT 3 h***

**Fig. 4:**
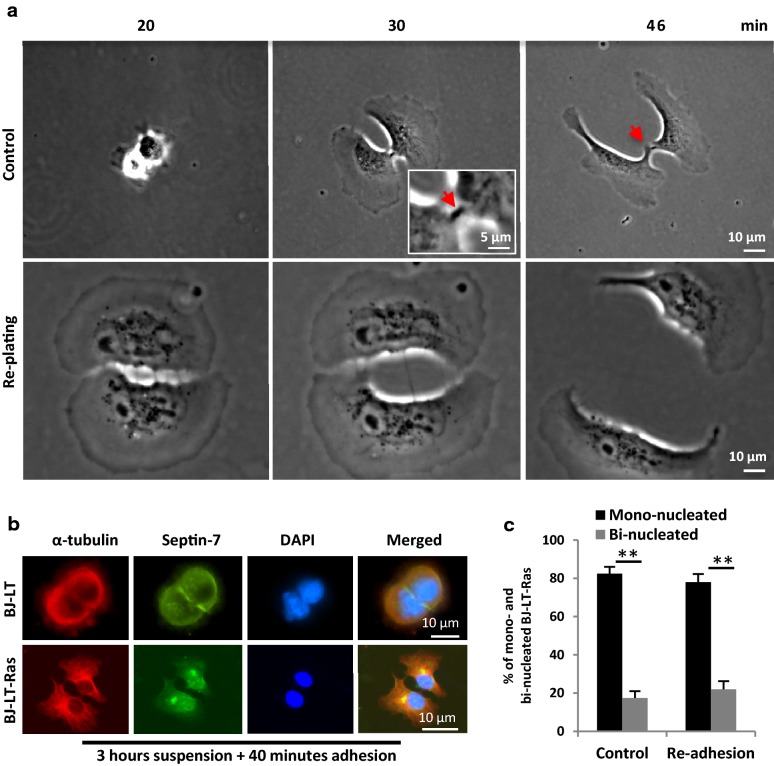
Re-adhesion reveals that active Ras promotes abscission in the detached cells. Mitotic BJ-LT and BJ-LT-Ras cells were isolated and either seeded directly on fibronectin (control) or cultured in suspension for 3 h before re-plating on fibronectin-coated coverslips. **a** Bright field micrographs from representative time-lapse movies showing the separation of two daughter BJ-LT-Ras cells at the indicated time points during re-adhesion to fibronectin directly (upper panel) or after 3 h suspension culture (lower panel); the latter cell is larger because of growth during the 3-h period. **b**, **c** Representative immunofluorescence micrographs illustrating the distribution of septin-7 (green) and α-tubulin (red) in BJ-LT-Ras (**b**) and BJ-LT after re-plating on fibronectin for 40 min with a pre-incubation for 3 h in suspension. Nuclei were stained with DAPI (blue). **c** % of cells that were separated from each other after 40 min re-plating as shown in **b** and after adhesion during the entire period of 3 h + 40 min (control cells)

We recently found that the distribution of septin-7 can be used as a marker to determine if abscission has taken place or not [15]. To investigate if such analysis of septin-7 and/or its interaction partner anillin could clarify if non-adherent BJ-LT-Ras cells can divide, the three cell lines were immune-stained for these proteins at different time points after isolation in M-phase and subsequent incubation under the adherent and non-adherent conditions (Fig. 3). Both proteins were localized at the ICB after 1 h in BJ cells under both culture conditions. In the divided adherent cells (3 h), anillin was not detectable and septin-7 was distributed throughout the cytoplasm (Fig. 3a). In the non-adherent BJ cells septin-7 remained in the MB region for > 24 h in most cells while anillin became diffusely localized to the nucleus (Fig. 3a, b). The enrichment of septin-7 between the two nuclei was found only in association with the ingressed plasma membrane at the ICB, and it was not due to cell–cell contacts as demonstrated by the staining of confluent cell cultures (Additional file 11: Figure S4). Essentially the same results were found for BJ-LT cells, i.e. septin-7 remained at the ICB under non-adherent conditions (Fig. 3c, d) in the absence of anillin and CEP55. Interestingly, most of the BJ-LT-Ras cells (80%) lacked enrichment of septin-7 in the contact area between the cells after 3 h in suspension, indicating that abscission had occurred (Fig. 3e, f). To further test this conclusion, the BJ-LT-Ras cells were re-plated on fibronectin after a 3-h period in suspension to analyze if and how the cells would separate. BJ daughter cells were previously shown to under these conditions be connected by the septin-stabilized ICB without midbody for 6–9 h after re-plating on fibronectin until the bridge is eventually broken by tension (cytofission) in the absence of MB (i.e. no ESCRT-mediated abscission) [15]. Here BJ-LT cells were found to behave in the same way. In contrast, BJ-LT-Ras cells rapidly and smoothly migrated apart without stretching of an ICB-like connection between the daughter cells (i.e. not by cytofission), and most cells were clearly separated already 20–40 min after re-plating (Fig. 4a–c, Additional file 12: Movie S8). Furthermore, the separation was faster also than abscission in the presence of MB under normal adherent conditions (Additional file 13: Movie S9). These data strongly support the conclusion that an ESCRT-mediated abscission was completed during the previous suspension period in BJ-LT-Ras cells.

### Septin persistently prevents regression of the ICB in the detached non-transformed fibroblasts

**Fig. 5:**
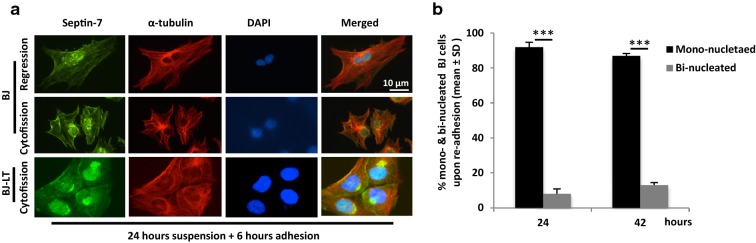
Regression of the ICB occurs in a small number of detached cells. M-cells were isolated and cultured in suspension for different time periods before re-plating on fibronectin-coated coverslips. **a** Representative fluorescence images showing the distribution of septin-7 (green) and α-tubulin (red) in BJ and BJ-LT after re-plating on fibronectin-coated surface for 6 h with a pre-incubation for 24 h in suspension. Note that tetra-lobular BJ-LT cells formed after 24 h in suspension (shown in Fig. 2) divided into four cells by cytofission after 6 h on fibronectin. **b** Mean% ± SD of the number of BJ cells that were separated from each other after incubation for 24 or 42 h in suspension followed by 6 h on fibronectin

As shown in Fig. 3, the rearrangement of septin-7 away from the ICB was slow in non-adherent BJ and BJ-LT cells, and the ring structure was maintained in > 80% of BJ cells after 42 h in suspension (Fig. 3a, b). However, at this time point the staining was reduced and the number of cells lacking the septin ring increased gradually with time. The BJ-LT cells had septin-7 located between all four lobes after 24 h in suspension (Fig. 3c, d), and at the 42-h time point the staining pattern was too complex to analyze since the cells had undergone yet another cell cycle round resulting in 8-nuclei cells which were folded into a cluster as seen by live-cell imaging (Additional file 7: Movie S4, Additional file 8: Movie S5). MB-independent cytofission occurred in most of the BJ and BJ-LT cells when they were re-plated on fibronectin for 6 h after prolonged times in suspension (Fig. 5a, b); only a small number of bi-nucleated cells was formed by regression of the ICB, which closely correlated with the numbers of cells lacking the septin-7 ring before re-plating (Fig. 3b, d). These observations confirm that septin can stabilize the ICB for > 40 h with high efficiency, however, a small number of potentially oncogenic bi-nucleated cells was generated after such extended time in suspension culture.

### Bi-nucleated BJ cells are halted in the G1 phase while BJ-LT become tetraploid

**Fig. 6:**
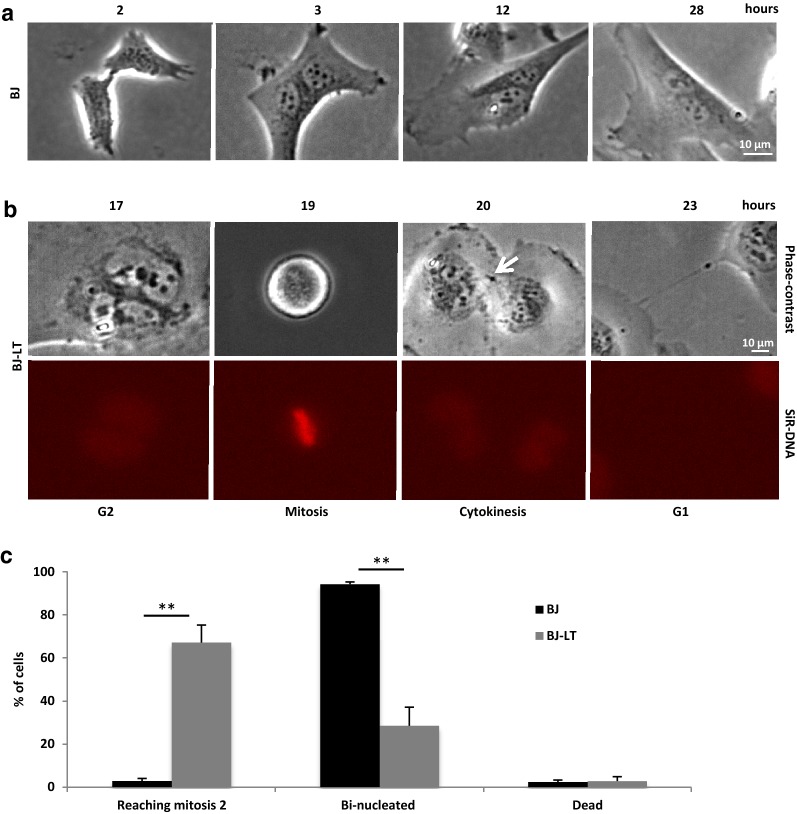
Bi-nucleated BJ cells are halted in the cell cycle while BJ-LT become tetraploid. M-cells were isolated and exposed to 0.5 µM CytD for 1 h as described under adhesion bi-nucleation model (Additional file 1: Figure S1), and then followed by live-cell imaging. **a** Bright field micrographs from time-lapse movie illustrating a single BJ mitotic cell failing in cytokinesis due to furrow regression induced by CytD. The formed bi-nucleated cells did not progress to the next mitosis as seen by the absence of mitotic cell-rounding. **b** Representative phase contrast and fluorescence micrographs from time-lapse movie of a single BJ-LT mitotic cell after CytD-induced cytokinesis failure. The bi-nucleated cells progressed to the next mitosis as seen by cell-rounding and formation of metaphase plate (at 19 h), followed by cytokinesis (20 h) and abscission (23 h). DNA was stained by SiR-DNA (red). The white arrow marks the midbody. **c** Mean% ± SD% of cells that proceeded to mitosis 2, remained binucleated, or died

To investigate the fate during the next cell cycle of bi-nucleated cells formed by failed cytokinesis, mitotic BJ and BJ-LT cells were isolated by shake off, re-plated on fibronectin-coated surface, and incubated with cytochalasin D (CytD) for 1 h (Additional file 1: Figure S1). By this treatment, a mixture of mononucleated cells and bi-nucleated cells was formed in the cultures, reflecting the different stages of mitosis present in the cell populations at the shake off step. CytD induced regression of the cleavage furrow in the cells which had not formed a MB at the time of drug exposure, whereas abscission was completed in cells that had reached far in the cytokinesis process when actin polymerization was inhibited by the drug [4]. Live-cell imaging showed that the bi-nucleated BJ cells did not round up for a new mitosis but remained bi-nucleated for several days, while mononucleated cells in the same cell population divided (Fig. 6a, c, Additional file 14: Movie S10). In contrast, bi-nucleated BJ-LT cells progressed to mitosis, aligned all chromosomes in metaphase, and divided into two mono-nucleated (4N) cells (Fig. 6b, c, Additional file 15: Movie S11).

**Fig. 7:**
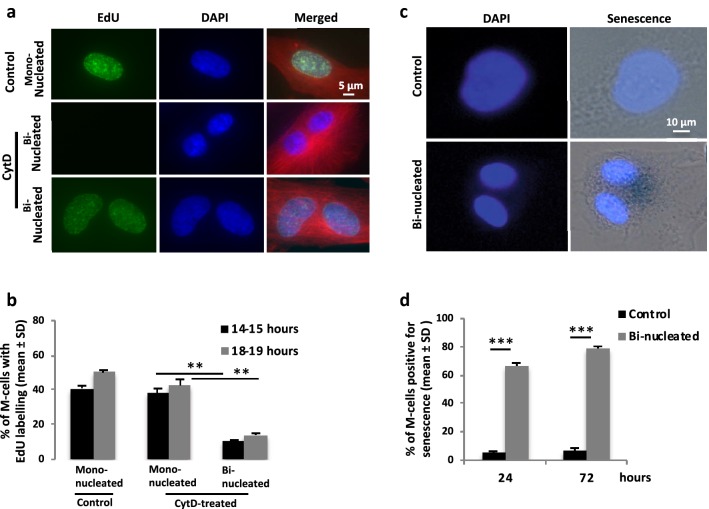
Adherent bi-nucleated BJ cells are halted in the G1 phase and become senescent. **a** Representative immunofluorescence images illustrating the absence and presence of EdU incorporation in DNA of mono- and bi-nucleated BJ cells cultured with and without CytD-treatment (described in Additional file 1: Figure S1). **b** Mean% ± SD of the isolated mitotic BJ cells that had progressed into the S phase of the following cell cycle at the indicated time points. Difference significance for CytD-treated: Mono-nucleated vs. Bi-nucleated**. **c** Representative phase-contrast images illustrating the absence and presence of X-gal staining (i.e. β-galactosidase activity; senescence marker) in BJ cells after previous treatment with and without CytD (described in Additional file 1: Figure S1). Note the large nucleus in the control cell compared to the senescent cells, reflecting their presence in the S and G1 phase, respectively. **d** Mean% ± SD of the isolated mitotic cells that show positive signal for senescence at the indicated time points of culture. Difference significance: Control vs. Bi-nucleated***

To determine at what stage in the cell cycle the bi-nucleated BJ cells were halted, entrance into S-phase was tested by a DNA synthesis assay. Mitotic cells released from CytD were incubated on fibronectin for 13 or 18 h followed by an additional hour in the presence of the nucleotide analogue EdU. For comparison, the mitotic cells not exposed to CytD were incubated with EdU at the same time periods. In the control condition (mono-nucleated cells), the number of EdU-positive cells increased with the incubation time while only a small fraction of the CytD-treated bi-nucleated cells were positive for EdU. CytD-exposed mono-nucleated cells in the same culture dish had a similar fraction of EdU-labelled nuclei as the control cells (Fig. 7a, b). These results show that BJ cells failing in cytokinesis did not progress into S-phase.

### Bi-nucleated BJ cells halted in the G1 phase become senescent

The non-proliferating bi-nucleated BJ cell could either have been halted in G1 or entered G0. These alternatives were distinguished by analysis for the presence of senescent cells in the population. The cells were treated with CytD for 1 h as described above and then cultured on fibronectin-coated glass for 24 or 72 h without CytD. A beta-galactosidase activity assay was performed, and the accumulation of perinuclear blue color was evaluated. For comparison, mitotic cells not exposed to CytD were analyzed in parallel. This analysis revealed that a large fraction of the bi-nucleated cells were positive for the senescence marker, having distinct blue color already after 24 h, which had further increased to 80% of the cells at the 72-h time point. As expected, only few control cells (5%) stained positively for senescence (Fig. 7c, d). This result shows that the adherent bi-nucleated BJ cells halted in the G1.

**Fig. 8:**
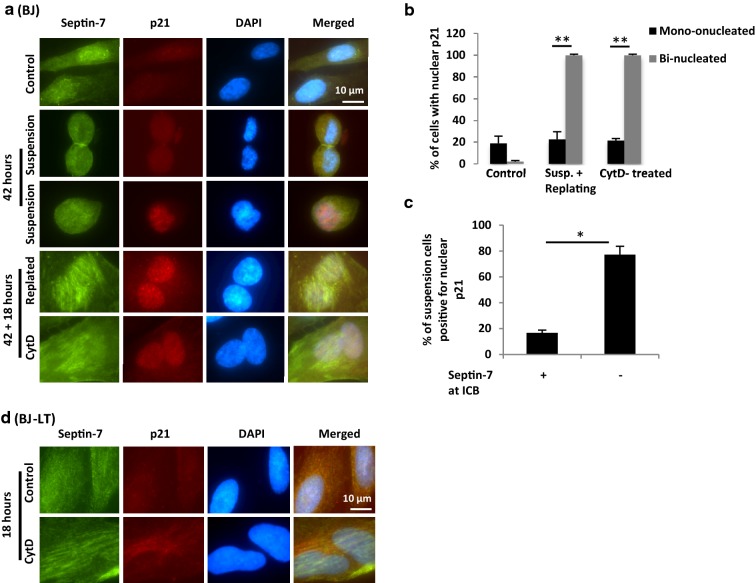
Bi-nucleated BJ cells with regressed ICB accumulate nuclear p21. Representative immunoflourescence images illustrating the distribution of septin-7 (green) and p21 (red) in mono- and bi-nucleated BJ and BJ-LT cells [also labelled for DNA (blue)]. **a** The isolated mitotic BJ cells were either seeded directly on fibronectin for 42 + 18 h (control), cultured in suspension for 42 h, or cultured in suspension for 42 h before re-plating on fibronectin-coated coverslips for 18 h. Alternatively, cells seeded on fibronectin were incubated for 1 h with or without CytD and further incubated for 42 + 18 h without CytD. **b** Mean% ± SD of BJ cells with positive staining for p21 in the nucleus under the conditions described in **a**. **c** Mean% ± SD of the BJ cells with positive staining for nuclear p21 in cell having or lacking septin-7 at the ICB after 42 h in suspension culture. **d** Mitotic BJ-LT cells seeded on fibronectin were incubated for 1 h with or without CytD and further incubated for 18 h without CytD

A main mechanism for induction of G1 senescence is the p53-dependent expression of p21. Bi-nucleated regressed BJ cells formed by suspension culture (Fig. 8a, rows 3 and 4) or by CytD-treatment under adherent conditions (Fig. 8a, bottom row) were found to accumulate p21 in the nuclei, whereas nuclear p21 was detectable in only few mononucleated cells in both cases (Fig. 8b). Notably, bi-nucleated non-regressed BJ cells from the suspension culture, i.e. cells with septin-7 at the ICB, were in most cases negative for nuclear p21 (Fig. 8a, row 2, c). In contrast, bi-nucleated BJ-LT cells formed by CytD-treatment under adherent conditions (Fig. 8d, bottom row) did not accumulate p21 as expected. These cells were fixed and stained 18 h after adhesion, a few hours before they would reach the next mitosis according to video recordings (Additional file 2: Figure S2E).

### Centrosomes fuse after cleavage furrow regression

**Fig. 9:**
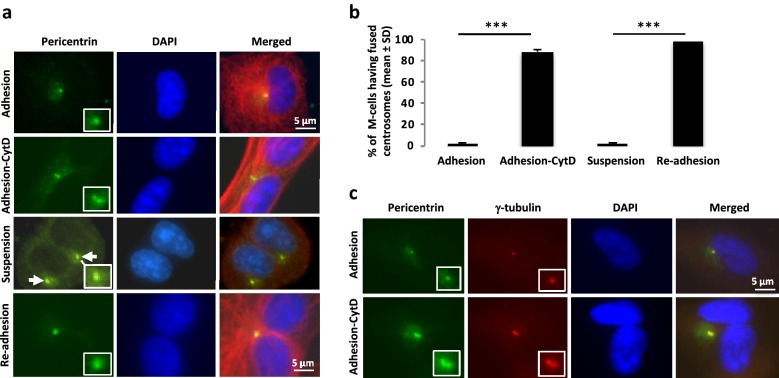
Centrosomes fuse after cleavage furrow regression. **a** Representative immunofluorescence images illustrating the distribution of the centrosome protein pericentrin (green) in mono- and bi-nucleated BJ cells [also labelled for α-tubulin (red) and DNA (blue)] cultured either on fibronectin-coated surface or in suspension (described in Additional file 1: Figure S1). The isolated mitotic cells were incubated on fibronectin for 1 h with or without CytD and fixed after further incubation for 2.5 h without CytD. Alternatively, binucleated cells formed by suspension culture for either 3.5 h, or 42 h followed by re-adhesion for 3 h on fibronectin, were stained as described above, and the regressed cells were analyzed. Upper row: mononucleated cell with one centrosome after completed cytokinesis; second row: binucleated regressed cell with two merged centrosomes; third row: cell with two nuclei and maintained ICB after 3.5 h in suspension having two separated centrosomes (white arrows); lower row: binucleated regressed cell after 42 h in suspension + 3 h on fibronectin with two merged centrosomes. **b** Mean% ± SD of the cells having fused centrosomes under the conditions described in **a**. Difference significance: Adhesion vs. Adhesion-CytD***, Suspension vs. Re-adhesion***. **c** Representative immunofluorescence micrographs illustrating co-localisation of pericentrin (green) and γ-tubulin (red) in the fused centrosomes. The square frames show the midbody region at higher magnification

Activation of the PIDDosome complex by merging of two centrosomes has recently been described as a mechanism by which bi-nucleated cells can activate p53-induced G1 senescence [19]. We analyzed the centrosome distribution by immune-staining for pericentrin and γ-tubulin in BJ cells which had become bi-nucleated either through furrow regression after CytD treatment or by suspension culture. As shown in Fig. 9a–c, newly divided cells had one centrosome as expected, while in 85% of CytD-treated regressed cells the two centrosomes had merged within 2.5 h after the drug washout. In detached cells which had failed in cytokinetic abscission, the two centrosomes remained separated at the same time point, presumably because the ICB was too narrow to allow centrosome passage (Fig. 9a, b). The minor fraction of regressed cells formed after prolonged suspension culture (42 h) followed by re-adhesion on fibronectin for 3 h had two closely merged centrosomes (Fig. 9a, bottom row).

## Discussion

To determine whether oncogenic HRas can promote cytokinesis in detached cells several methods were tested. First, analysis of ALIX recruitment to the MB in the detached cells revealed that BJ-LT-Ras cells can pass the step which is blocked in non-adherent BJ and BJ-LT cells. By live-cell imaging, some of the BJ-LT-Ras cells appeared to progress all the way to the completed division, as judged by their different morphology compared to BJ and BJ-LT cells and the distinct light diffraction by the membrane between the two associated cells. However, under non-adherent conditions the abscission plane is not in perfect focus of the microscope in all cells, and therefore this method does not accurately monitor the proportion of divided cells. As an alternative approach, the distribution of septin and anillin was analyzed. The septin filament system is known to undergo reorganization and redistribution according to different cell activities [15, 20], and anillin localization has been used as a marker for the different stages of the cell cycle [21]. In this study anillin was found to disappear from the ICB in non-adherent cells after prolonged incubation, presumably by proteolytic degradation at G1 entrance [21]. Consistent with previous reports, new anillin synthesized later in interphase was accumulated and stored in the nucleus until mitosis [22]. Thus, anillin did not provide information about abscission. However, the presence of septin-7 between the two nuclei may indeed be a useful marker for uncut ICB; it remained located at this area for > 24 h after isolation of M-cells in 90% of the non-adherent BJ and BJ-LT cells while it was redistributed into the cytoplasm in 80% of non-adherent BJ-LT-Ras cells within 3 h. These numbers correlate well with the results obtained after re-adhesion on fibronectin of cells previously kept in suspension. The re-adhering BJ and BJ-LT cells could not divide by the MB-dependent abscission mechanism since the MB had dissolved after incubation in suspension and do not reform until next mitosis since key proteins are either degraded (e.g. Aurora B, PLK1, anillin) or redistributed (e.g. CEP55) during interphase. However, 90% of them separated by cytofission due to rupture of the narrow ICB that was maintained by septin; this type of cell division required 5–6 h of adhesion in agreement with previous observations [15]. In contrast, most of the BJ-LT-Ras cells were present as mono-nucleated cells which had clearly migrated apart already 20–40 min after re-adhesion on fibronectin, strongly indicating that abscission had occurred during the suspension period.

At present the mechanism is unknown by which HRas promotes the recruitment of ALIX to CEP55 and the completion of abscission independently of integrin signals. Our previously reported results showed that inhibitors of PI3 K, MEK, ROCK, or PLC3 do not markedly affect these late steps in cytokinesis [4], and further work is needed to identify the signaling mediators downstream of HRas that are involved in cytokinesis regulation. Such work is highly motivated since the molecular mechanisms presumably promote colony formation from single cells, a distinguishing property of malignant tumor cells, and detailed knowledge of the process may reveal potential targets for tumor treatment.

Bi-nucleated cells are thought to be a possible cause of cancer because they may give rise to tetraploid cells, which are genomically unstable. However, for cells failing at abscission due to detachment from the extracellular matrix, septin serves as a protection mechanism against formation of bi-nucleated cells according to our data. Septin assembled at the ICB stabilizes and efficiently prevents regression of the narrow structure, and septin thereby promotes cytofission (midbody-independent cell division) if the cells would re-adhere. However, the septin organization at the ICB slowly disappears and the number of regressed, bi-nucleated cells therefore increases over time. Thus, the mechanism for septin disassembly is an important topic that needs to be clarified. Fortunately, such bi-nucleated cells can be prevented to proliferate due to p53/p21-dependent control system(s), as shown by the comparison of BJ- and BJ-LT cells (Fig. 8). Adherent bi-nucleated BJ cells, formed by blocking the cytokinesis with a short exposure (1 h) to CytD, did not enter a new S-phase and were halted in G1 as senescent cells. In contrast, the adherent bi-nucleated BJ-LT cells progressed to a new mitosis where both karyokinesis and cytokinesis were successful in most of the cells, and thereby mono-nucleated tetraploid cells were formed (Fig. 6, Additional file 15: Movie S11). Thus, such potentially tumor-causing cells can be produced if transiently detached cells also have a suppressed p53 response, a situation that may exist during certain virus infections.

The existence of a so-called tetraploid checkpoint has been a controversial issue for a long time [23]. Recent reports strongly support the conclusion that some tetraploid states, e.g. bi-nucleated cells, actually do induce a p53 response and G1 arrest, but that it is due to the presence of more than one mature centrosome rather than the number of chromosomes. Two mature centrosomes in G1 cells were shown to bind to each other via their appendage structures and thereby trigger the assembly of the PIDDosome protein complex, which activates p53 via caspase 2-mediated degradation of Mdm2 [19]. A second p53-activating mechanism linked to > 1 centrosome in G1 was reported to use the Hippo tumor suppressor pathway [23]. In our study, the two centrosomes in adherent bi-nucleated BJ cells rapidly merged after the failed cytokinesis, indicating that the G1 arrest and senescence were induced by the PIDDosome mechanism. Bi-nucleated cells in suspension maintaining an ICB had two separate centrosomes as expected considering the narrow space in the ICB. These cells were not senescent since after re-plating on fibronectin, they instead underwent cytofission and then proceeded to a new mitosis. The different outcomes identified in this study of normal and transformed cells that lose the integrin-mediated adhesion are summarized in Additional file 16: Figure S5.

## Conclusions

This study demonstrates that several mechanisms contribute to prevent detached normal cells from generating tumor-causing tetraploid cells, including blocked cytokinesis abscission when they are not adherent, cytofission if they re-adhere, and G1 arrest/senescence of bi-nucleated cells. In particular, an important role for septin to prevent furrow regression was found. However, tetraploid cells can be produced if transiently detached cells also have a suppressed p53 response, e.g. by viruses, mutation or inflammation. The second major finding is that the expression of an activating Ras mutation can overcome the abscission block in non-adherent transformed cells. This ability promotes colony growth in vitro, and possibly also the growth of tumor metastasis.

## Materials and methods

### Cell lines and culturing of mitotic cells

The cell lines BJ (hTERT-immortalized human non-transformed fibroblast cells), BJ-LT (SV40 large T-antigen (LT)-transfected BJ cells), BJ-LT-Ras (BJ-LT cell transfected with oncogenic HRas mutant) [24] were cultured in Dulbecco’s modified Eagle medium (DMEM, Gibco, Life technologies, UK) supplemented with 10% fetal bovine serum (FBS, FB-1090–500, Werner Saveen, Biological Industries, Beit-Haemek Ltd, Israel), 100 U/ml penicillin and 0.1 mg/ml streptomycin (complete medium). The cells were kept at 37 °C in a humid atmosphere containing 5% CO_2_. In some experiments 0.5–5 μM cytochalasin D (CytD, Sigma-Aldrich) were used to induce binucleation. Mitotic cells were collected by the shake off method in which exponentially growing cells were washed once with pre-warmed PBS, followed by incubation in complete medium for approximately 2–3 h, and then the loosely attached mitotic cells were detached by tapping the culture flasks. The cells were collected and re-suspended in fresh complete medium for culturing in either ultra-low attachment plates or on plates coated with fibronectin (40 μg/ml). Since BJ-LT cell had a higher tendency to aggregate via cell–cell contacts, 1.2% methylcellulose (M-7027, Sigma-Aldrich) was included in the culture medium when these cell lines were cultured in suspension in ultra-low attachment wells.

### Live-cell imaging

Live-cell imaging was performed using an inverted microscope (Nikon-Eclipse Ti-U, Japan) equipped with a CCD camera (Andor’s multi pixel sCMOS camera, Oxford Instruments) and a cell culture chamber having constant supply of humidified 5% CO_2_ and temperature control. The images were acquired using an automated motorized multi-position stage with 20× and 40× magnification objectives and phase contrast filter of the time-lapse microscope in 2–15 min time intervals for the desired time periods. Adherent cells were monitored in fibronectin-coated culture plates whereas non-adherent cells were monitored in 6-well ultra-low attachment plates (catalog no. 10154431, Thermo Fisher Scientific, Sweden) in the presence of 1.2% methylcellulose. In some experiments, SiR-Hoechst (SiR-DNA; Spirochrome) at a 1:1000 dilution was used as a DNA marker for live-cell imaging.

### Immunofluorescence staining

For the adhesive condition, the mitotic cells were cultured on fibronectin-coated glass coverslips, whereas for the non-adhesive condition they were cultured in 6-well ultra-low attachment plates (catalog no. 10154431, Thermo Fisher Scientific, Sweden) and thereafter the cells were deposited on glass slides by cytospin centrifugation. Subsequently, the cells were fixed by cold methanol at − 20 °C for 20 min and then washed twice in PBS for 5 min. After incubation in blocking buffer containing 1% BSA (Fraction V Roche Diagnostic, Germany) and 0.1% Tween20 (Merck, Germany) in PBS, the samples were incubated overnight at 4 °C with primary antibodies at a 1:50 dilution in the blocking buffer. Antibodies directed against the following proteins were used: Aurora B (ab 3609, ab 2254, Abcam), CEP55 (ab 170414, Abcam), CHMP4B (sc-82556, Santa Cruz) p21 (Clone 70/Cip1/WAF1, BD biosciences), α-tubulin (T6199, Sigma, Saint Louis, USA), Septin-7 (JP18991, IBL International, Hamburg, Germany), Anillin (sc-271814, Santa Cruz), γ-tubulin (sc-17787, Santa Cruz), pericentrin (ab4448, rabbit polyclonal, Abcam, Cambridge, UK). The glasses were then washed with PBS and incubated for 1 h with a 1:500 dilution in the blocking buffer of the secondary antibodies Alexa Fluor 488-conjugated goat anti-rabbit and Alexa Fluor 594-conjugated goat anti-mouse (Invitrogen, Carlsbad, USA), followed by washing in PBS and mounting with medium containing DAPI (4,6-diamidino-2-phenylindole, Invitrogen). Digital images of the cells were captured using a Nikon fluorescence microscope (Nikon Eclipse 90i, Japan) equipped with a CCD camera (DS-Qi1 Monochromatic Digital Camera). The digital images were analysed for the presence or absence of immunostained proteins at specific locations and scored using Adobe Photoshop© (Adobe Photoshop CS6, Adobe system Inc. San Jose, CA, USA) and ImageJ (http://rsb.info.nih.gov) software.

### Quantification of cytokinesis failure/tetraploid cells

Upon plating on fibronectin-coated substrate, the isolated round mitotic cells flattened, divided, and migrated away from each other. Successful cell division could be clearly identified when they had moved apart, and since the migrating cells frequently made transient contacts with each other, time-lapse imaging allowed the most accurate quantification of divided cells. In the fixed samples, the cells were scored after staining for microtubules (MT), septin-7 and DNA as regressed, divided, or ingressed but failed in abscission.

### EdU incorporation analysis

EdU detection was performed according to the protocol (Click-iT EdU Imaging Kit, C10084, Invitrogen Molecular Probes, Eugene, Oregon, USA). The cells were incubated with 10 μM EdU for 1 h prior to fixation with 4% formaldehyde and incubation with the EdU detection reagent. In addition, the cells were immunostained for MT and images were acquired in the fluorescence microscope to analyse EdU incorporation together with tubulin staining.

### β-Galactosidase staining as marker for cell senescence

Mitotic BJ cells were plated on fibronectin-coated coverslips, treated with or without CytD (0.5–5 μM for 1 h), and then washed two times with pre-warmed PBS and two times with pre-warmed complete medium at 5 min intervals. Cells were then cultured for 24–48 h in a CO_2_ incubator followed by staining for β-galactosidase according to the manufacture of the β-galactosidase staining kit for senescence (#9860, Cell signaling). The slides were mounted with DAPI as mounting medium and the cells were photographed using the Nikon Eclipse 90i fluorescence microscope.

### Statistical analysis

The statistical analyses were performed using Student’s *t*-test. *P*-values < 0.05 were considered as significant. For all the experiments, 40–50 randomly selected cells per condition and for each time point were analysed from each of three independent experiments. ***, **, and * represent *P*-value less than 0.001, 0.01 and 0.05, respectively.

## Additional files

**Additional file 1: Figure S1.** Schematic description of the experimental design. Mitotic (M) cells collected by the shake off method were analyzed as illustrated above in two different models whereby bi-nucleated cells are formed by cytokinesis failure. In the suspension model, two daughter cells are connected via an ICB after failure at a late cytokinesis stage close to abscission. These cells were cultured in ultra-low attachment plates for varying times and then used for live-cell imaging or immunofluorescence. In the adhesion model, M-cells were directly re-plated on fibronectin and incubated with or without CytD for 1 h to prevent or allow formation of the ingression furrow at the beginning of cytokinesis, respectively, and then analyzed as indicated in the figure.

**Additional file 2: Figure S2.** Kinetics of cytokinesis and cell cycle progression in BJ-LT and BJ-LT-Ras. (A, B) Representative immunofluorescence images illustrating the presence of Aurora B, CEP55, and α-tubulin at the intercellular bridge (ICB) in BJ-LT and BJ-LT-Ras after adhesion of isolated mitotic cells to fibronectin for 1 h. (C) Mean% ± SD of cells progressing to cytokinesis. (D) Mean% ± SD of cells completing cytokinetic abscission during the indicated time intervals after adhesion to fibronectin as analysed by live-imaging. (E) Mean% ± SD of cells completing one cell cycle within the indicated time intervals after adhesion to fibronectin as analysed by live-imaging.

**Additional file 3: Movie S1.** The video shows the progression of cytokinesis from furrow ingression to the abscission in BJ-LT cells adhering to fibronectin. A red arrow in the first frame of the movie indicates a cytokinetic cell.

**Additional file 4: Movie S2.** The video shows the progression of cytokinesis from furrow ingression to the abscission in BJ-LT-Ras cells adhering to fibronectin.

**Additional file 5: Figure S3.** Active Ras, but not SV40 LT protein, promotes tubulin bundling and the recruitment of CHMP4B to the MB in detached cells. Representative immunofluorescence images illustrating the presence of α-tubulin (red) and CHMP4B (green) at the ICB and MB, respectively, in mitotic BJ-LT (A) and BJ-LT-Ras cells (C) after culture for 1 h on fibronectin or in suspension. (B, D) Mean% ± SD of the number of cells having CHMP4B at the MB. The square frames show the midbody region at higher magnification.

**Additional file 6: Movie S3.** The video shows the appearance of a mitotic BJ cell after failed cytokinesis under non-adherent condition. The bi-nucleated cell maintains the cytokinetic furrow ingression and does not show furrow regression during the 24-h period.

**Additional file 7: Movie S4.** These videos shows the appearance of mitotic BJ-LT cells after failed cytokinesis under non-adherent condition. The bi-nucleated cell progressed into next mitosis in the cell cycle and does not show the furrow regression. Movie S4 is indicated with two examples. A cytokinetic cell is indicated with a red arrow in the first frame and the bi-lobular cell progressed into tetra- and multi-lobar cells after approximately one and two cell cycle doubling time periods, respectively. For example, the 41 h 20 min time point shows multi-lobular structures (red arrows).

**Additional file 8: Movie S5.** These videos shows the appearance of mitotic BJ-LT cells after failed cytokinesis under non-adherent condition. The bi-nucleated cell progressed into next mitosis in the cell cycle and does not show the furrow regression. Another cytokinetic cell (red arrow) showing similar features as the cell in movie.

**Additional file 9: Movie S6.** These videos illustrate the process of cytokinesis in BJ-LT-Ras cells under non-adherent condition. The bi-nucleated cell progresses from furrow ingression to apparent abscission within 1.5 h from the isolation M-cells, as suggested by the morphology and the diffraction light pattern at the membrane between two emerged daughter cells; the daughter cells remain associated at the 3 h time point.

**Additional file 10: Movie S7.** These videos illustrate the process of cytokinesis in BJ-LT-Ras cells under non-adherent condition. Another cytokinetic cell showing similar features as the cell in movie.

**Additional file 11: Figure S4.** Septin is not enriched at cell–cell borders in confluent BJ cells. Confluent BJ cells were stained for septin-7 (green) and anillin (red). Nuclei were stained with DAPI (blue).

**Additional file 12: Movie S8.** These videos illustrate the indirect confirmation that BJ-LT-Ras induces adhesion-independent cytokinesis. A mitotic BJ-LT-Ras cell re-plated on fibronectin-coated surface after a 3-h period in suspension. Note that cells migrate apart without presence of an ICB.

**Additional file 13: Movie S9.** These videos illustrate the indirect confirmation that BJ-LT-Ras induces adhesion-independent cytokinesis. For comparison, this video shows the progression of cytokinesis from furrow ingression in a mitotic BJ-LT-Ras cell under control conditions, i.e. seeded on fibronectin directly after isolation.

**Additional file 14: Movie S10.** The video illustrates the fate of a mitotic BJ cells after treatment with 0.5 µM cytochalasin D for 1 h, followed by 25 h without drug. Four cells were marked with a red arrow in the first frame. One of the marked cells (to the right) exhibits furrow regression (becomes bi-nucleated) and does not reach to next mitosis; in one cell (to the left) the furrow ingression is inhibited (becomes bi-nucleated) and does not reach to next mitosis; the two other marked cells (at the top) divide (become mono-nucleated) and reach to next mitosis.

**Additional file 15: Movie S11.** The video illustrates the fate of a mitotic BJ-LT cells after treatment with 0.5 µM cytochalasin D for 1 h, followed by 25 h without drug in the presence of 1:1000 SiR-DNA. A cell marked with red arrow in the first frame becomes bi-nucleated by furrow regression, later reaches to next mitosis, and divides as two tetraploid cells.

**Additional file 16: Figure S5.** A schematic description for the fate of bi-nucleated cells generated by cleavage furrow regression or abscission failure. In the adhesion bi-nucleated model, BJ cells regress the cleavage furrow in the early cytokinesis (C) stage during CytD treatment and become arrested in the next G1-phase (2 + 2N DNA and two centrosomes). The centrosomes merge, which may promote PIDDosome assembly and stabilization of p53 leading to cell senescence. In contrast, BJ-LT fibroblasts progress to S- and M-phase (8N (nuclei marked dark blue) and 4 centrosomes) and most of them form a bi-polar spindle (possibly by clustered centrosomes (marked dark brown)) to segregate their duplicated chromosomes and generate tetraploid cells. In the suspension bi-nucleation model, bi-nucleated cells are connected by an intercellular bridge (ICB) containing a midbody (MB) in the center, and each prospective daughter cell has one nucleus and one centrosome. When entering the G1-phase, the MB is dissolved in BJ cells under the non-adherent condition without completion of abscission, but the ICB is stabilized by septin for longer time. BJ fibroblasts are halted in G1-phase due to lack of integrin signals, consistent with their non-transformed nature, and and keep the bi-lobular structure. BJ-LT cells instead progress into S- and M-phase and become tetra-lobular due to suppression of the G1/S checkpoint by the SV40/LT protein. BJ-LT-Ras cells under the same condition complete abscission, but often remain associated by cell–cell contacts. Upon re-adhesion to a fibronectin surface, these BJ-LT-Ras cells quickly migrate apart, which confirm the completion of abscission under the previous non-adherent period. For the re-adhering BJ and BJ-LT cells the lobular structures become separated from each other after a longer time (traction-based abscission, cytofission). Note that under re-adhesion condition, a few regressed bi-nucleated cells are present (shown in the red block), which can further develop in the same way as described in the Adhesion bi-nucleated model.

## Data Availability

All data generated or analyzed in this study are included in the article.

## Abbreviations

CytD: cytochalasin D
DAPI: 4,6-diamidino-2-phenylindole
DMEM: Dulbecco’s modified Eagle medium
FBS: fetal bovine serum
ICB: intercellular bridge
LT: large T antigen
MB: midbody
MT: microtubule
