# Supplementary material for: Septin and Ras regulate cytokinetic abscission in detached cells
Source: Cell Div. 2019 Aug 21;14:8. doi: 10.1186/s13008-019-0051-y (PMC6702736; doi:10.1186/s13008-019-0051-y)
Supplement: Supplementary file 1 — Additional file 1: Figure S1. Schematic description of the experimental design. Mitotic (M) cells collected by the shake off method were analyzed as illustrated above in two different models whereby bi-nucleated cells are formed by cytokinesis failure. In the suspension model, two daughter cells are connected via an ICB after failure at a late cytokinesis stage close to abscission. These cells were cultured in ultra-low attachment plates for varying times and then used for live-cell imaging or immunofluorescence. In the adhesion model, M-cells were directly re-plated on fibronectin and incubated with or without CytD for 1 h to prevent or allow formation of the ingression furrow at the beginning of cytokinesis, respectively, and then analyzed as indicated in the figure. [file 13008_2019_51_MOESM1_ESM.pptx]

## Slide 1
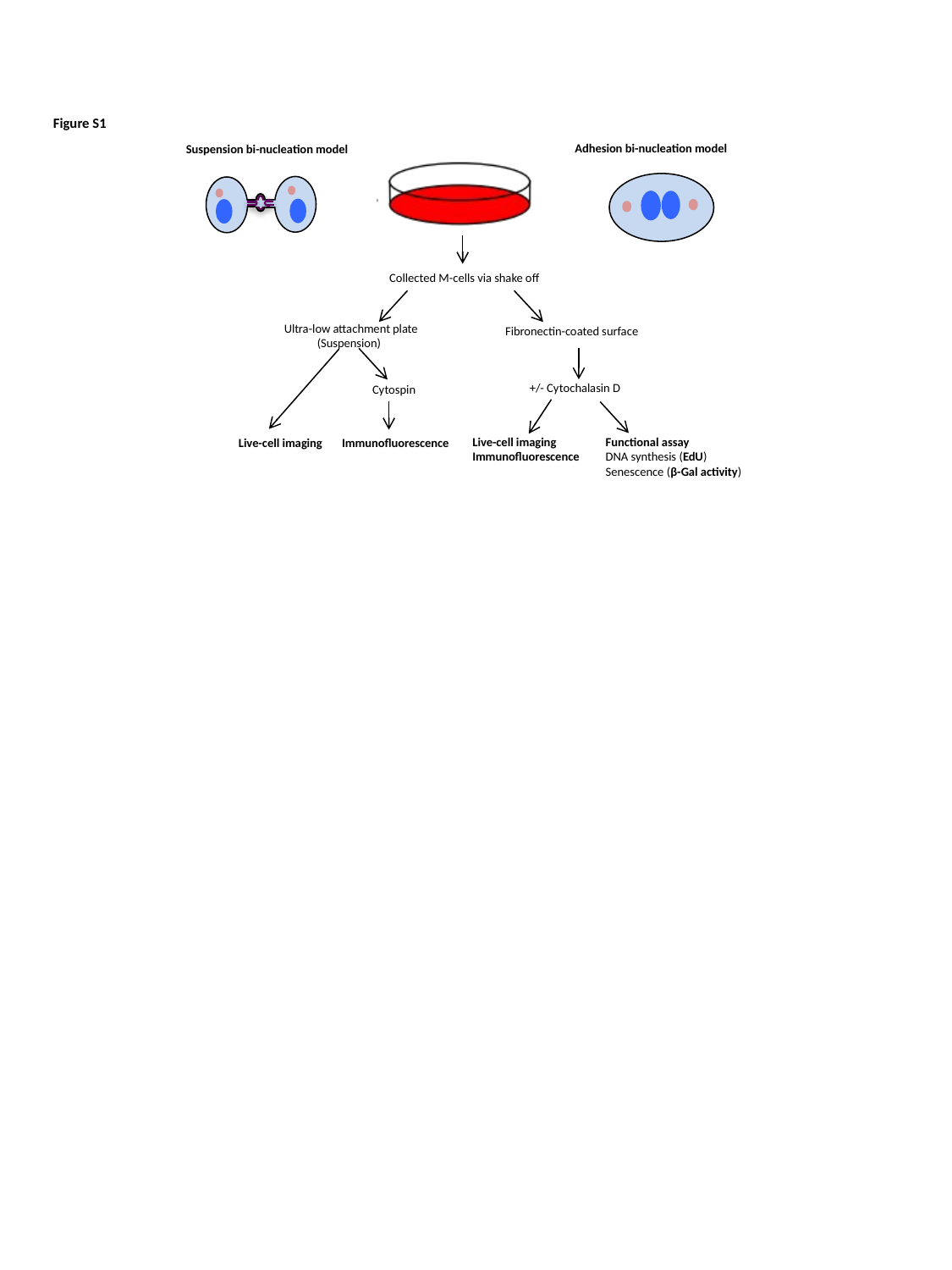

Figure S1
Adhesion bi-nucleation model
Suspension bi-nucleation model
Collected M-cells via shake off
 Ultra-low attachment plate
 (Suspension)
 Fibronectin-coated surface
+/- Cytochalasin D
Cytospin
Functional assay
DNA synthesis (EdU)
Senescence (β-Gal activity)
Live-cell imaging
Immunofluorescence
Live-cell imaging
Immunofluorescence
