# Supplementary material for: Septin and Ras regulate cytokinetic abscission in detached cells
Source: Cell Div. 2019 Aug 21;14:8. doi: 10.1186/s13008-019-0051-y (PMC6702736; doi:10.1186/s13008-019-0051-y)
Supplement: Supplementary file 2 — Additional file 2: Figure S2. Kinetics of cytokinesis and cell cycle progression in BJ-LT and BJ-LT-Ras. (A, B) Representative immunofluorescence images illustrating the presence of Aurora B, CEP55, and α-tubulin at the intercellular bridge (ICB) in BJ-LT and BJ-LT-Ras after adhesion of isolated mitotic cells to fibronectin for 1 h. (C) Mean% ± SD of cells progressing to cytokinesis. (D) Mean% ± SD of cells completing cytokinetic abscission during the indicated time intervals after adhesion to fibronectin as analysed by live-imaging. (E) Mean% ± SD of cells completing one cell cycle within the indicated time intervals after adhesion to fibronectin as analysed by live-imaging. [file 13008_2019_51_MOESM2_ESM.pptx]

## Slide 1
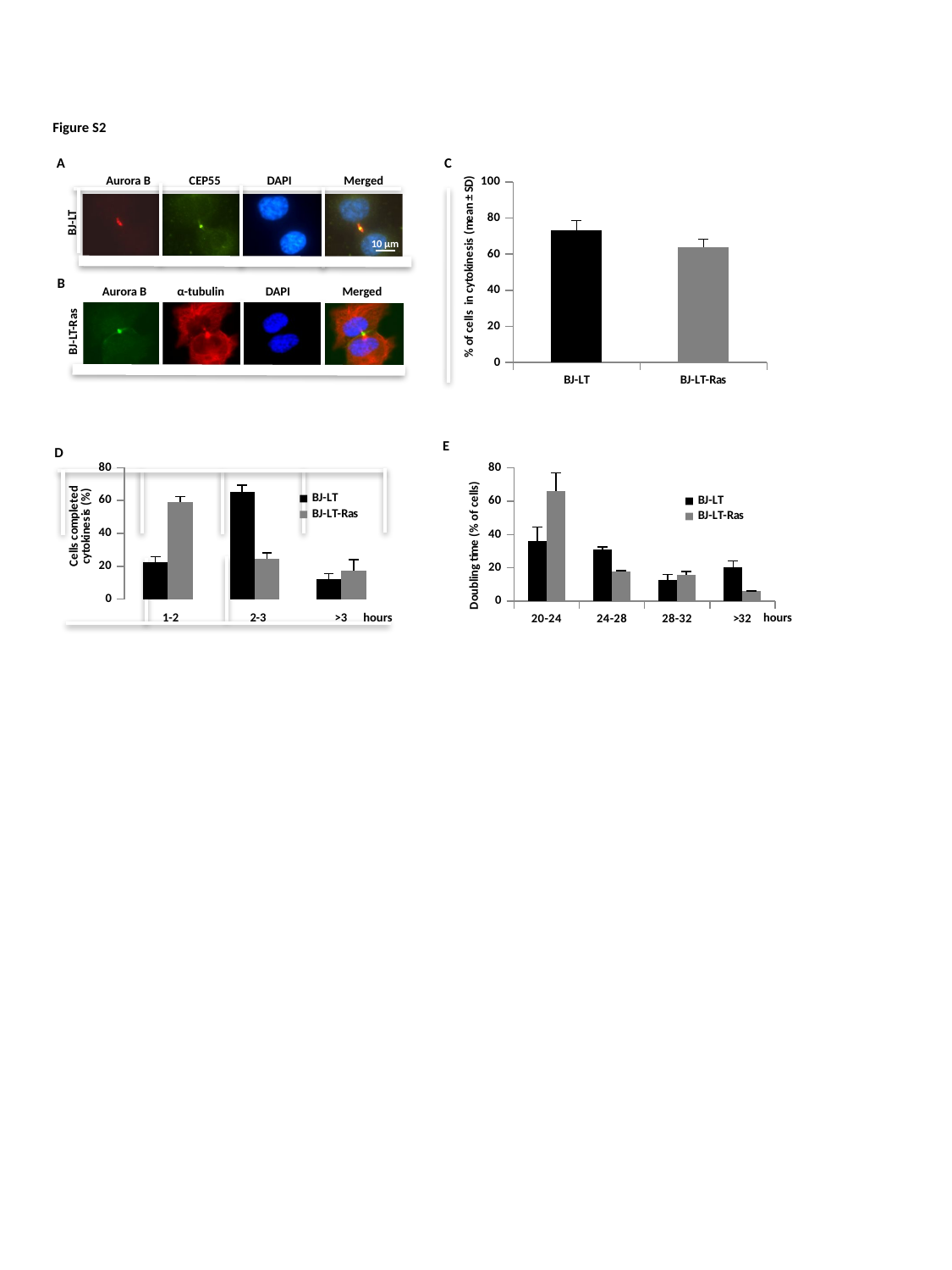

Figure S2
A
C
### Chart
| Category | |
|---|---|
| BJ-LT | 73.0 |
| BJ-LT-Ras | 64.0 |Aurora B CEP55 DAPI Merged
10 μm
BJ-LT
B
Aurora B α-tubulin DAPI Merged
BJ-LT-Ras
### Chart
| Category | BJ-LT | BJ-LT-Ras |
|---|---|---|
| 20-24 | 36.0 | 66.16999999999997 |
| 24-28 | 31.0 | 17.75 |
| 28-32 | 12.5 | 15.85 |
| >32 | 20.5 | 5.9 |E
D
### Chart
| Category | BJ-LT | BJ-LT-Ras |
|---|---|---|
| 1-2- | 22.4 | 59.11333333333334 |
| 2-3- | 65.4 | 24.33333333333328 |
| >3 | 12.2 | 17.33333333333328 |hours
1-2 2-3 >3 hours
