# Supplementary material for: Septin and Ras regulate cytokinetic abscission in detached cells
Source: Cell Div. 2019 Aug 21;14:8. doi: 10.1186/s13008-019-0051-y (PMC6702736; doi:10.1186/s13008-019-0051-y)
Supplement: Supplementary file 5 — Additional file 5: Figure S3. Active Ras, but not SV40 LT protein, promotes tubulin bundling and the recruitment of CHMP4B to the MB in detached cells. Representative immunofluorescence images illustrating the presence of α-tubulin (red) and CHMP4B (green) at the ICB and MB, respectively, in mitotic BJ-LT (A) and BJ-LT-Ras cells (C) after culture for 1 h on fibronectin or in suspension. (B, D) Mean% ± SD of the number of cells having CHMP4B at the MB. The square frames show the midbody region at higher magnification. [file 13008_2019_51_MOESM5_ESM.pptx]

## Slide 1
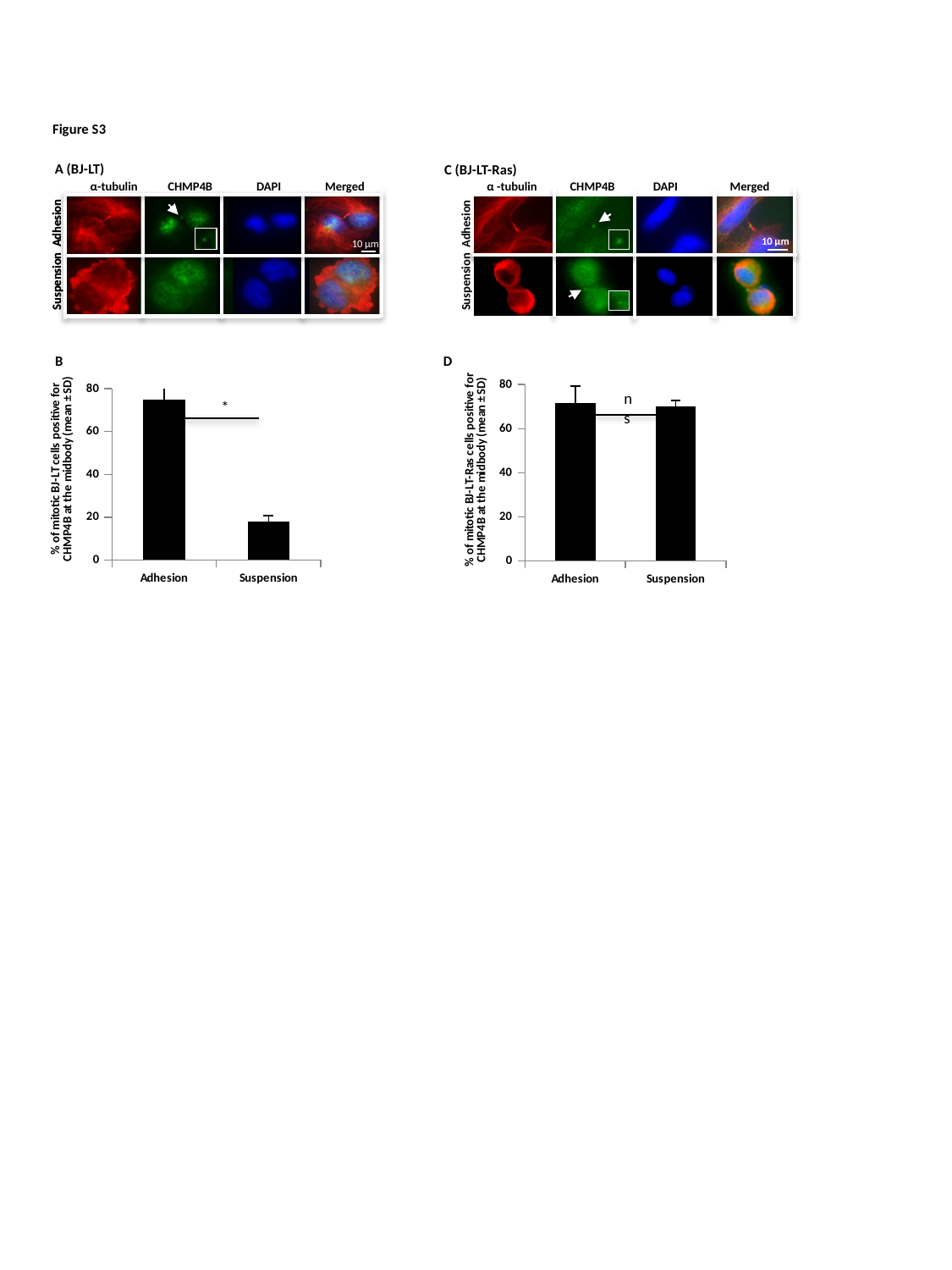

Figure S3
A (BJ-LT)
C (BJ-LT-Ras)
α -tubulin CHMP4B DAPI Merged
10 μm
Suspension Adhesion
 α-tubulin CHMP4B DAPI Merged
10 μm
Suspension Adhesion
Suspension Adhesion
B
D
### Chart
| Category | CHMP4B |
|---|---|
| Adhesion | 75.0 |
| Suspension | 18.0 |
### Chart
| Category | Average |
|---|---|
| Adhesion | 71.5 |
| Suspension | 70.0 |*
ns
