# Supplementary figures and images for: Septin and Ras regulate cytokinetic abscission in detached cells
Source: Cell Div. 2019 Aug 21;14:8. doi: 10.1186/s13008-019-0051-y (PMC6702736; doi:10.1186/s13008-019-0051-y)

## Slide 1
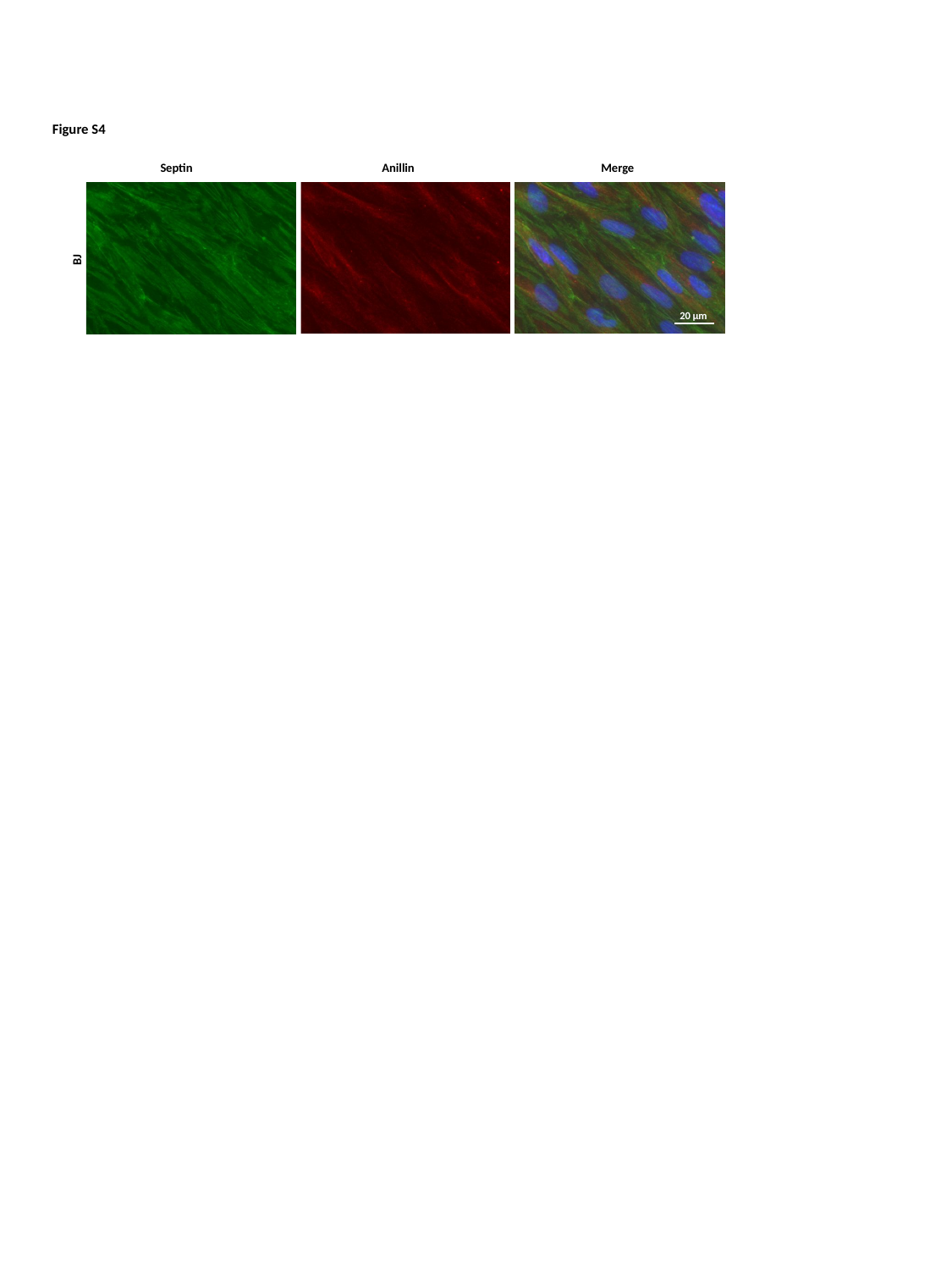

Figure S4
Septin Anillin Merge
BJ
20 μm

Supplement: Supplementary file 11 — Additional file 11: Figure S4. Septin is not enriched at cell–cell borders in confluent BJ cells. Confluent BJ cells were stained for septin-7 (green) and anillin (red). Nuclei were stained with DAPI (blue). [file 13008_2019_51_MOESM11_ESM.pptx]
