# Supplementary material for: Septin and Ras regulate cytokinetic abscission in detached cells
Source: Cell Div. 2019 Aug 21;14:8. doi: 10.1186/s13008-019-0051-y (PMC6702736; doi:10.1186/s13008-019-0051-y)
Supplement: Supplementary file 16 — Additional file 16: Figure S5. A schematic description for the fate of bi-nucleated cells generated by cleavage furrow regression or abscission failure. In the adhesion bi-nucleated model, BJ cells regress the cleavage furrow in the early cytokinesis (C) stage during CytD treatment and become arrested in the next G1-phase (2 + 2N DNA and two centrosomes). The centrosomes merge, which may promote PIDDosome assembly and stabilization of p53 leading to cell senescence. In contrast, BJ-LT fibroblasts progress to S- and M-phase (8N (nuclei marked dark blue) and 4 centrosomes) and most of them form a bi-polar spindle (possibly by clustered centrosomes (marked dark brown)) to segregate their duplicated chromosomes and generate tetraploid cells. In the suspension bi-nucleation model, bi-nucleated cells are connected by an intercellular bridge (ICB) containing a midbody (MB) in the center, and each prospective daughter cell has one nucleus and one centrosome. When entering the G1-phase, the MB is dissolved in BJ cells under the non-adherent condition without completion of abscission, but the ICB is stabilized by septin for longer time. BJ fibroblasts are halted in G1-phase due to lack of integrin signals, consistent with their non-transformed nature, and and keep the bi-lobular structure. BJ-LT cells instead progress into S- and M-phase and become tetra-lobular due to suppression of the G1/S checkpoint by the SV40/LT protein. BJ-LT-Ras cells under the same condition complete abscission, but often remain associated by cell–cell contacts. Upon re-adhesion to a fibronectin surface, these BJ-LT-Ras cells quickly migrate apart, which confirm the completion of abscission under the previous non-adherent period. For the re-adhering BJ and BJ-LT cells the lobular structures become separated from each other after a longer time (traction-based abscission, cytofission). Note that under re-adhesion condition, a few regressed bi-nucleated cells are present (shown in the red block), which [file 13008_2019_51_MOESM16_ESM.pptx]

## Slide 1
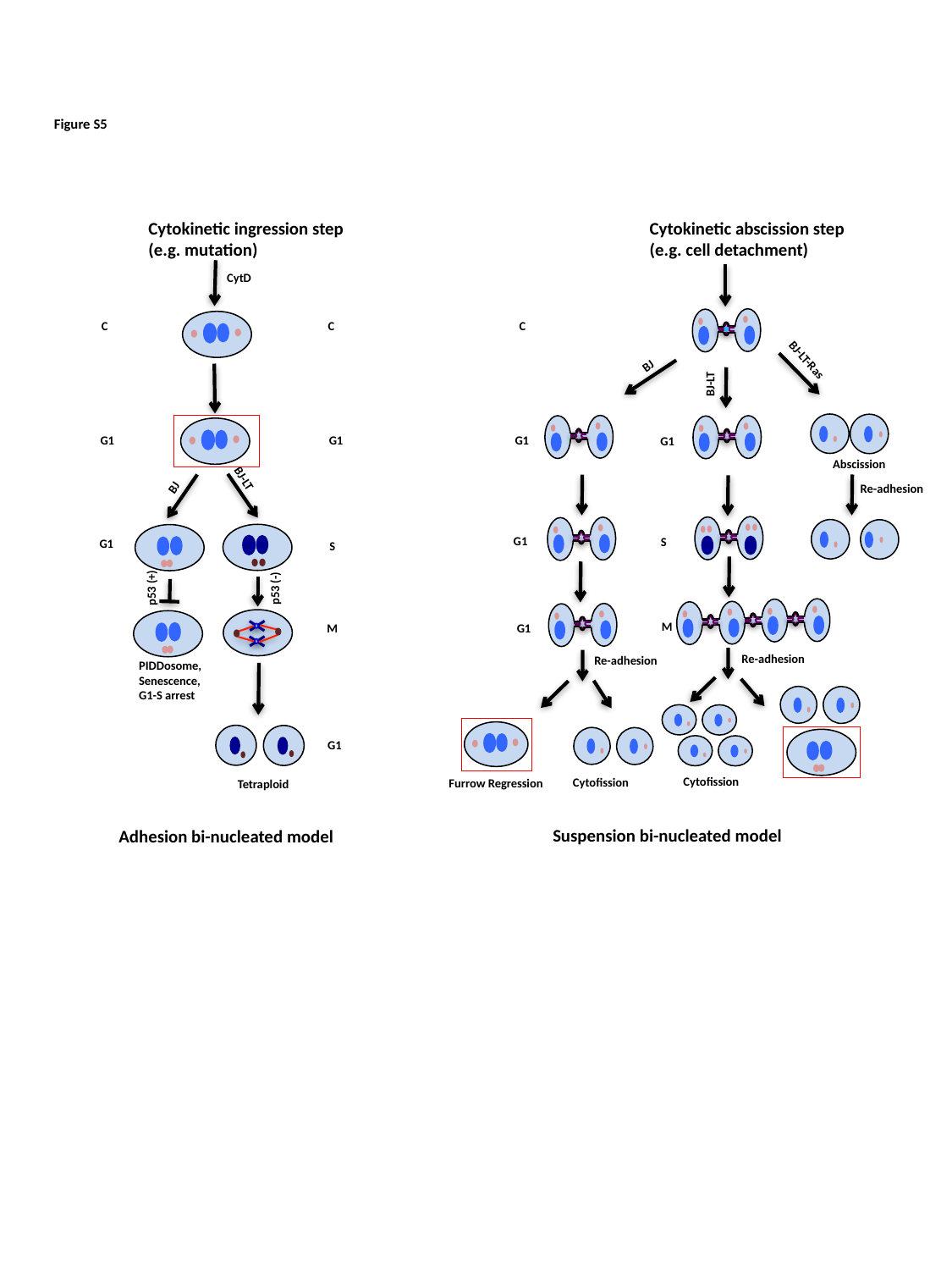

Figure S5
Cytokinetic ingression step
(e.g. mutation)
Cytokinetic abscission step
(e.g. cell detachment)
CytD
C
C
C
BJ-LT-Ras
BJ
BJ-LT
G1
G1
G1
G1
Abscission
BJ-LT
BJ
Re-adhesion
G1
S
G1
S
p53 (+)
p53 (-)
M
M
G1
Re-adhesion
Re-adhesion
PIDDosome,
Senescence,
G1-S arrest
G1
Cytofission
Cytofission
Furrow Regression
Tetraploid
Suspension bi-nucleated model
Adhesion bi-nucleated model
